# Identification of Atrial Fibrillation-Associated Genes *ERBB2* and *MYPN* Using Genome-Wide Association and Transcriptome Expression Profile Data on Left–Right Atrial Appendages

**DOI:** 10.3389/fgene.2021.696591

**Published:** 2021-06-30

**Authors:** Xiangguang Meng, Yali Nie, Keke Wang, Chen Fan, Juntao Zhao, Yiqiang Yuan

**Affiliations:** ^1^Laboratory of Cardiovascular Disease and Drug Research, Zhengzhou No. 7 People’s Hospital, Zhengzhou, China; ^2^Department of Pharmacology, School of Medicine, Zhengzhou University, Zhengzhou, China; ^3^Skin Research Institute of Singapore, Agency for Science, Technology and Research (A^∗^STAR), Singapore, Singapore; ^4^Department of Cardiac Surgery, Zhengzhou No. 7 People’s Hospital, Zhengzhou, China; ^5^Department of Cardiovascular Internal Medicine, Henan Provincial Chest Hospital, Zhengzhou, China

**Keywords:** atrial fibrillation, genome-wide association study, single nucleotide polymorphism, systems biology, transcriptome

## Abstract

More reliable methods are needed to uncover novel biomarkers associated with atrial fibrillation (AF). Our objective is to identify significant network modules and newly AF-associated genes by integrative genetic analysis approaches. The single nucleotide polymorphisms with nominal relevance significance from the AF-associated genome-wide association study (GWAS) data were converted into the GWAS discovery set using ProxyGeneLD, followed by merging with significant network modules constructed by weighted gene coexpression network analysis (WGCNA) from one expression profile data set, composed of left and right atrial appendages (LAA and RAA). In LAA, two distinct network modules were identified (blue: *p* = 0.0076; yellow: *p* = 0.023). Five AF-associated biomarkers were identified (*ERBB2*, *HERC4*, *MYH7*, *MYPN*, and *PBXIP1*), combined with the GWAS test set. In RAA, three distinct network modules were identified and only one AF-associated gene *LOXL1* was determined. Using human LAA tissues by real-time quantitative polymerase chain reaction, the differentially expressive results of *ERBB2*, *MYH7*, and *MYPN* were observed (*p* < 0.05). This study first demonstrated the feasibility of fusing GWAS with expression profile data by ProxyGeneLD and WGCNA to explore AF-associated genes. In particular, two newly identified genes *ERBB2* and *MYPN* via this approach contribute to further understanding the occurrence and development of AF, thereby offering preliminary data for subsequent studies.

## Introduction

Atrial fibrillation (AF), manifesting as irregular AF, is the most common arrhythmia in clinical practice and leads to many serious complications such as stroke, heart failure, ventricular arrhythmias, and peripheral arterial embolism (Barrios et al., 2012). Epidemiological investigations have reported that the prevalence of AF dramatically increases with age (Bapat et al., 2018). AF causes a high risk of disability and death (Acciarresi et al., 2017), which places a heavy burden on individuals and the healthcare system. It has been reported that the underlying mechanisms of the occurrence and maintenance of AF are regulated by genetic factors, particularly single nucleotide polymorphisms (SNPs) (He et al., 2018; Lin et al., 2019). Therefore, the accurate identification of SNPs is essential for the investigation of AF-associated genes.

To date, genome-wide association study (GWAS) has been the most popular method for SNP identification and has been widely used in the study of various complex diseases due to its high throughput and cost-effectiveness. In particular, some mutations associated with AF have been identified using GWAS by setting a standard threshold *p*-value > 5E–08 (Jannot et al., 2015; Christophersen et al., 2017; Nielsen et al., 2018; Roselli et al., 2018). However, this arbitrary threshold discards many mutations with subtle effects but potential biological significance (*p* > 5E–08), which in turn leads to a partial loss of heritability (Jia et al., 2012). Thus, network analysis methods, such as pathway enrichment analysis, are required to solve the missing heritability problem (Askland et al., 2009; Elbers et al., 2009).

Gene ontology (GO) and the Kyoto Encyclopedia of Genes and Genomes (KEGG), two representative enrichment analysis approaches, are designed based on hypergeometric distribution. They function by comparing the number of interesting genes enriched in a special term or pathway with the number of random background genes. Although these methods are user-friendly, their statistical efficiency is limited since expressive information of genes is not taken into account (Farber, 2013). Therefore, the more powerful gene-set enrichment analysis (GSEA), combined with the signal strength of gene expression, was applied for pathway analysis of genes in this study.

Studies have demonstrated that the weighted gene coexpression network analysis (WGCNA), another pathway analysis software, has the characteristics of reliable robustness and repeatability for expression profile data, and can obtain the interconnections between genes and find the network modules relative to diseases using gene expression values (Zhang et al., 2017; Kong et al., 2019; Wang et al., 2019). It is reported that one network-integrated approach combining GWAS with expression profile data by WGCNA possesses significant advantages in mining hidden disease-associated pathways and functional genes, compared with other existing approaches. Farber (2013) used this analysis method to repeat the previous functional pathways from osteoporosis-associated GWAS and transcriptome data, and identify newly functional genes. Chen et al. (2016) validated the potential ability of this method by exploring the hidden biological functions in a larger data set associated with osteoporosis. The joint method, however, has not been applied for AF-associated studies.

In this study, we converted SNPs with nominal relevance significance from the AF-associated GWAS data into the GWAS discovery set using ProxyGeneLD, followed by merging with the AF-associated modules constructed by WGCNA, and finally obtained two newly AF-associated biomarkers *ERBB2* and *MYPN*. This study offers a systemic analytical method for revealing hidden AF-associated genes from GWAS and expression profile data.

## Materials and Methods

### Software Used

All analyses were conducted based on Cytoscape 3.7.2 (Shannon et al., 2003), GSEA 4.0.2 (Subramanian et al., 2005), LD score regression (LDSC) 1.0.1 (Bulik-Sullivan et al., 2015; Finucane et al., 2015), ProxyGeneLD (Hong et al., 2009), and R-related packages (R 3.6.1, 2019).

### Pre-processing of GWAS Data

The keyword “atrial fibrillation” was used for the GWAS catalog database and retrieved 24 data sets, in which GCST006414 (Nielsen et al., 2018) were pre-processed as the GWAS discovery set, followed by merging with expressive profile data. The data set included 1,030,836 European individuals and 34,740,186 SNPs with precomputed *p*-values. The raw data for the test set came from FinnGen research project Freeze 3 (Finngen_r3_I9_AF^1^), which included 86,200 European individuals and 16,932,622 SNPs with precomputed *p*-values. Either total heritability of the two GWAS summary_statistic datasets was calculated by LDSC. Please refer to the following detailed steps, https://github.com/bulik/ldsc/wiki/Heritability-and-Genetic-Correlation.

Based on linkage disequilibrium (LD), gene length, and variant locus density, the *p*-values of SNPs from the GCST006414 were converted into the adjusted *p*-values (adj *p*) of genes by ProxyGeneLD, whose reference genome was set to the genome reference consortium human genome build 37 (GRCh37). LD patterns were determined using HapMap LD data set hg19_2009.04_rel27 from CEU (Utah residents with Northern and Western European ancestry from the CEPH collection). LD threshold (*R*^2^) was set to a default value (>0.8), and the 5′ and 3′ ends of each gene sequence were recognized and extended by 2,000 and 1,000 bp, respectively. The correction of *p*-values was based on the false discovery rate (FDR). The ultimately retained genes made up the GWAS discovery set (adj *p*_dis_ < 0.05) and the GWAS test set (adj *p*_test_ < 0.05), respectively, for the following analysis.

### Pre-processing of Expression Profile Data

The keyword “atrial fibrillation” was used to retrieve the GEO database, and three data sets (GSE79768, GSE115574, and GSE128188) from individuals with the paired LAA and RAA samples were obtained. Considering that the races of the samples from GSE79768 and GSE115574 are not consistent with the GWAS data sets and that the relevant literature of GSE115574 has not yet been published, they were discarded. The GSE128188 selected is produced by next-generation RNA sequencing (Illumina NextSeq 500) and contains 10-paired LAA and RAA samples from five male patients in sinus rhythm (SR) (average age: 62.4 ± 6.87 years) and 5 male patients in AF (average age: 73.6 ± 5.12 years), of European descent. The two data sets were normalized to build up the LAA- and RAA-normalized express lists by the DESeq function of DESeq2 package in R environment. To determine whether there were outlier samples, the “plotPCA” function was used for principal component analysis (PCA).

### Constructing Network Modules by WGCNA

The correlation between each pair of the top 5,000 genes with high SDs in each of LAA- and RAA-normalized express lists was calculated to gain a correlation matrix, and then one adjacency matrix was obtained using a soft threshold (power index) instead of an arbitrarily hard threshold in an unsupervised manner. The power index was used to minimize the weaker connections and amplify the stronger connections between nodes in the matrix. The adjacency matrix was eventually used to calculate a topological overlap measure (TOM), and one dissimilarity matrix was obtained by 1 – TOM. The one-step network construction function “BlockwiseModules,” provided by the WGCNA package (Langfelder and Horvath, 2008), was applied to the construction of WGCNA cluster trees. Different branches of cluster tree represent different gene modules and are shown in different colors. The parameter “minModuleSize” was set to 30 to avoid modules with very few genes, and the “mergeCutHeight” parameter was set to 0.25 to combine genes with lower values. For details of the procedure, please refer to the website: https://horvath.genetics.ucla.edu/html/CoexpressionNetwork/Rpackages/WGCNA/Tutorials/.

### Description of Network-Related Parameters for WGCNA

Weighted gene coexpression network analysis generates multiple parameter metrics. Module eigengene (ME) represents the expression value of all genes in one module after dimensionality reduction by the first PCA, and it can be used to determine the biological significance of the module combined with external phenotypes. In this study, the correlation between ME and AF was determined by the contingency coefficient (CC), which was calculated from the ME-dichotomized values around the median by the assocstats function (Liu et al., 2015a,b), given that the disease status (AF or SR) is a dichotomous variable. Gene significance (GS) was defined as minus log 10 of the *p*-value obtained by the DESeq function, measuring differential expression between AF and SR groups, for individual genes among all modules (Farber, 2010). In addition, the association between GS and module membership (MM) was used to verify the relationship between ME and AF (Farber, 2013), and the threshold for significance is set to *p* < 0.05 and *R* > 0.3. MM was defined as the correlation of ME in one module and gene expression values. For example, the blue MM*_*i*_* = cor(xi, ME_blue_) measures how correlated the expression value of one gene *i* is to the blue ME. The blue MM*_*i*_* measures the membership of the *i*-th gene as regards the blue module. If the blue MM*_*i*_* is close to 0, then the *i*-th gene is not part of the blue module. But if the blue MM*_*i*_* is close to 1 or –1, it is highly associated with the blue module genes.

### Gene-Set Enrichment Analysis

Gene-set enrichment analysis v4.0.3 was applied to obtain the AF-associated pathways. The four human gene sets (c5.bp/cc/mf.v7.0.symbols.gmt and c2.cp.kegg.v7.0.symbols.gmt) from the Molecular Signatures Database (MsigDB, a collection of annotated gene sets for use with GSEA software) were chosen. In addition, the value of parameter “Permutation type” was set to “gene sets,” and the other parameters were set by default. The corrected *p*-value (FDR) for the screening criteria of function pathways is set to FDR < 0.25.

### Application of Cytoscape Software and Vennerable Package

First, two gene lists (GS > 2) are generated from AF-associated network modules constructed from LAA- and RAA-normalized express lists, respectively, which are taken as input data sets of the venn.diagram function in Vennerable package. The result image returned by this function can show the different distribution of coexpression pattern genes between LAA and RAA. Furthermore, according to weights between genes (>0.15), the two gene lists are input into Cytoscape for visualization to determine core genes under the conditions of adj *p*_dis_ < 0.05 and GS > 2, which are then validated by the GWAS test set (adj *p*_test_ < 0.05).

### Patient Samples

All 30 patients with valvular heart diseases who received cardiac surgery at Zhengzhou No. 7 People’s Hospital were enrolled and classified into permanent AF (*n* = 12) and SR (*n* = 18) groups. Written informed consents were obtained from all participants. AF rhythm status was documented by electrocardiogram for >3 months. Patients with mitral valve or mitral valve combined other valve disease, who had coronary heart disease and hypertension, were included. Patients with other arrhythmia or other diseases such as pulmonary disease, diabetes mellitus, hyperthyroidism, rheumatic disease, autoimmune disease, congenital heart disease, and myocardial bridge were excluded. The data of all patients are shown in Table 1. The LAA tissues of patients were snap frozen in liquid nitrogen and kept at –80°C until RNA extraction. All procedures with human were in accordance with the Declaration of Helsinki. The experimental scheme was approved by the ethics committee of Zhengzhou No. 7 People’s Hospital (Approval No. 20190804).

**TABLE 1:** Clinical characteristics of AF and SR groups.

**Clinical characteristics**	**AF (*n* = 12)**	**SR (*n* = 18)**	**OR**	***P*-value**	**95% CI**
Sex (male/female)	5:7	9:9	0.72	0.722	0.12, 3.90
Age (years)	61.42 ± 8.76	61.5 ± 8.17	–	0.979	–6.50, 6.67
Smoking (yes/no)	4/8	6/12	1	1	0.15, 5.97
Drinking (yes/no)	3/9	6/12	0.68	0.70	0.09, 4.30
MV/MV + OV	3/9	5/13	0.87	1	0.11, 5.92
CAD (yes/no)	12/0	18/0	–	–	–
HP (yes/no)	12/0	18/0	–	–	–
DM (yes/no)	0/12	0/18	–	–	–

*AF, atrial fibrillation; CAD, coronary artery disease; DM, diabetes mellitus; HP, hypertension; MV, mitral valve disease; MV + OV, mitral valve disease combined the other valve disease; SR, sinus rhythm.*

### Validation of Real-Time Quantitative Polymerase Chain Reaction

The differential expression genes using data-mining were validated in human LAA by real-time quantitative polymerase chain reaction (RT-qPCR). All primers are shown in Supplementary File 1. The expression level of each gene was detected by SYBR GREEN method using the StepOnePlus system (Applied Biosystems, Foster City, CA, United States), with the housekeeping GAPDH as the internal reference gene. The reaction system volume was 20 μl, and the reaction conditions were 95°C 60 s, then 95°C 5 s and 60°C 40 s, lasting 40 cycles. The Ct value is the cycle number reflecting to reach the detection threshold of fluorescence signal. Relative gene expression levels were calculated by 2^–ΔΔ*Ct*^. Mann–Whitney *U*-tests were used for significance difference analysis by ΔΔCt values, and the data were reported as mean ± SE. Amplifications were performed in triplicate for each sample.

## Results

### Overviews of GWAS Data Sets and Network Modules Constructed

**FIGURE 1:**
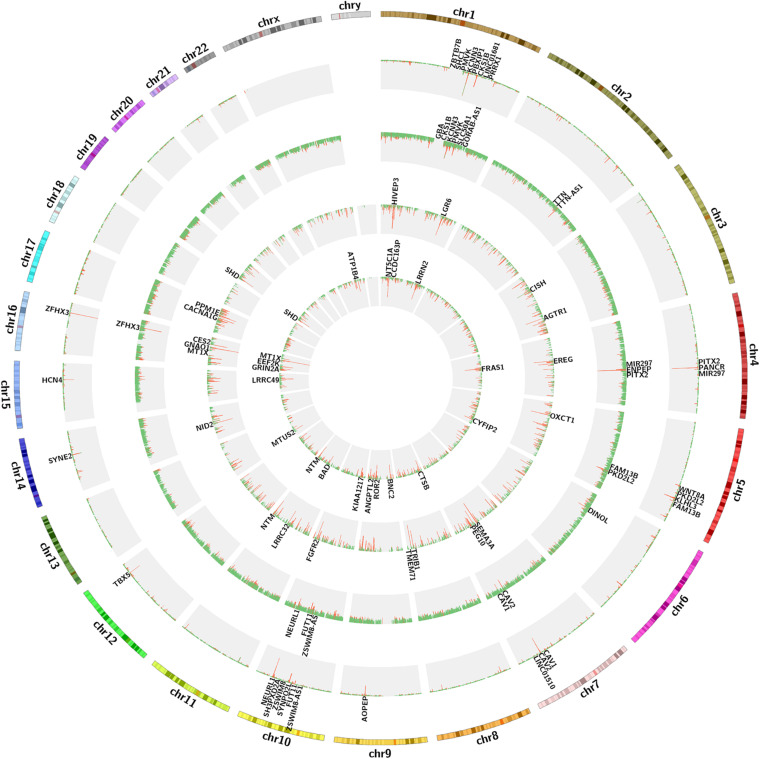
The circos diagram for GWAS and expression data sets. Two outermost tracks represent the chromosome text and banding positions. From the outside to the inside, the first and second histogram tracks represent the GWAS discovery and test sets, respectively. The red abundance value along the vertical axis represents the genes with adj *p* < 0.05, the first 20 of which are displayed in the first and second text tracks, and the green represents the genes with adj *p* > 0.05. The third and four histogram tracks represent the GS values of the expression sets of LAA and RAA, respectively. The red abundance value along the vertical axis represents the genes with GS > 2, the first 20 of which are displayed in the third and four text tracks, and the green represents the genes with GS < 2. GS, gene significance; LAA, left atrial appendage; RAA, right atrial appendage.

**FIGURE 2:**
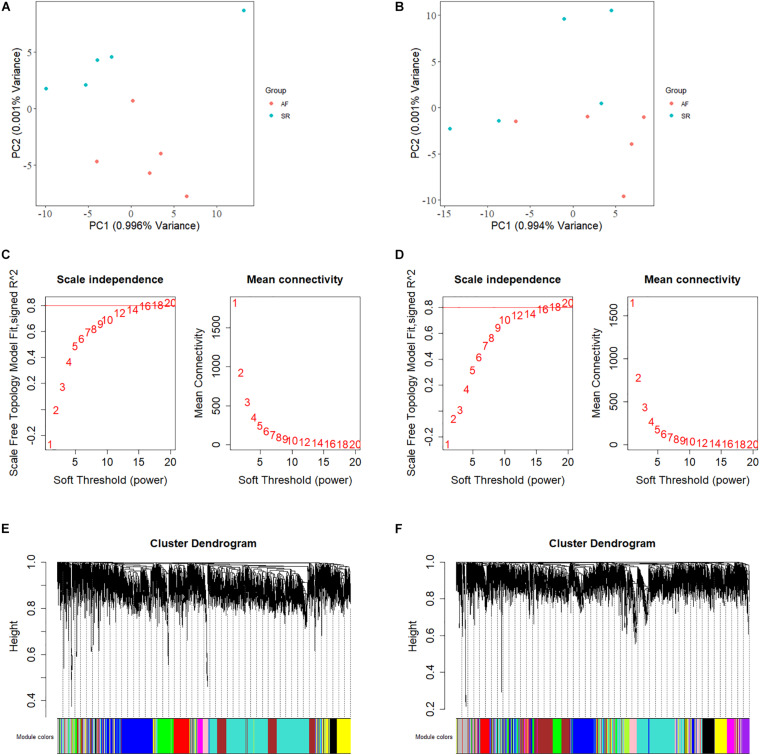
The principal component analysis, the calculation of power indexes, and the generation of module cluster trees on expression data sets. **(A)** The PCA plot is shown for determination of outlier samples in LAA. **(B)** The PCA plot is shown for determination of outlier samples in RAA. **(C)** Analysis of network topology for various soft-thresholding powers in LAA. The *y*-axis of the left panel represents the scale-free fit index while the *x*-axis indicates the soft-thresholding power. The *y*-axis of the right panel displays the mean connectivity and the *x*-axis also represents the soft-thresholding power. **(D)** Analysis of network topology for various soft-thresholding powers in RAA. **(E)** The construction of module cluster tree in LAA. In the picture, branches correspond to the coexpression modules with highly interconnected groups of genes. **(F)** The construction of module cluster tree in RAA. LAA, left atrial appendage; PCA, principal component analysis; RAA, right atrial appendage.

We calculated the total heritability/SE/intercept of two GWAS datasets in this study by LDSC and observed that there was little difference in heritability between the two microarrays (GCST006414: 0.0236/0.0022/1.0503 and Finngen_r3_I9_AF: 0.0485/0.0087/1.0324). Although their intercept values are close to 1, more data are needed to confirm how close they are to the true heritability because there are few variables available from the GWAS summary_statistic datasets such as environmental factors. The GWAS discovery and test sets were constructed by ProxyGeneLD, composed of 2,676 and 1,142 genes with adj *p* < 0.05, respectively (showed in red in the first and second histogram tracks of Figure 1), and the GS values of first 5,000 genes with high standard deviations from the LAA and RAA of GSE128188 are shown in the third and four histogram tracks of Figure 1. There were no outlier values observed in the expression sets of LAA and RAA (Figures 2A,B). Both WGCNA networks of LAA and RAA were constructed, and the power indexes β were confirmed as 18 using the “pickSoftThreshold” function (Figures 2C,D). Ten and 12 distinct gene modules were identified in LAA and RAA, respectively. A unique color was assigned to each module, and the ranges of the gene number of modules from LAA and RAA were from 101 (magenta) to 1,642 (turquoise), and from 159 (green yellow) to 1,066 (turquoise). Two gray modules with 448 and 634 genes represented the background color (Figures 2E,F).

### Discovery of the Modules With Best Biological Implications

**FIGURE 3:**
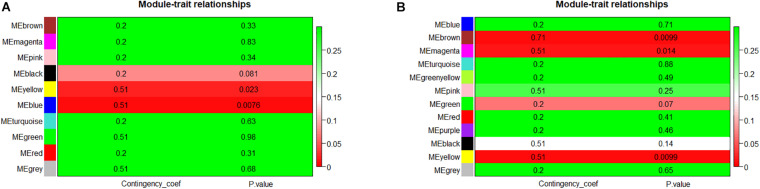
The module–trait associations of networks. **(A)** Module–trait associations in LAA. Row represents a module eigengene, column indicates disease status – AF or SR. Each cell contained the corresponding correlation and *p*-value. The table was color-coded by *p*-value according to the color legend. **(B)** Module–trait associations in RAA. LAA, left atrial appendage; RAA, right atrial appendage.

Module eigengene metrics corresponds to the first principal component of a given module and is considered as the most representative gene expression in module. The correlation between ME and AF was estimated by CC metrics. Significant results were displayed in the blue (CC = 0.51, *p* = 0.0076) and yellow (CC = 0.51, *p* = 0.023) modules of LAA, and in the yellow (CC = 0.51, *p* = 0.0099), brown (CC = 0.71, *p* = 0.0099), and magenta (CC = 0.51, *p* = 0.014) modules of RAA (Figures 3A,B).

**FIGURE 4:**
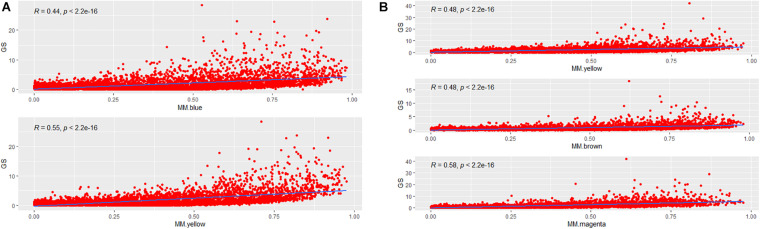
The correlation between MM and GS. **(A)** The correlation between MM and GS for each of two AF-associated modules in LAA. MM for each module is plotted against GS. **(B)** The correlation between MM and GS for each of three AF-associated modules in RAA. GS, gene significance; LAA, left atrial appendage; RAA, right atrial appendage.

To incorporate the AF trait into the network modules, we introduced GS measure. Theoretically, an increased GS value of the *i*-th gene leads to enhanced biological significance of the *i*-th gene. GS metrics captures the difference between AF and SR groups for each gene by *p*-values of hypothesis testing. MM measures how tightly a special gene fits into its module and thus reflects the module cohesiveness. Furthermore, the relationship between GS and MM verifies the biological significance of module. As shown in Figures 4A,B, significantly positive correlations were observed in the blue (*R* = 0.44, *p* = 2.2E–16) and yellow (*R* = 0.55, *p* = 2.2E–16) modules of LAA, and likewise showed in the yellow (*R* = 0.48, *p* = 2.2E–16), brown (*R* = 0.48, *p* = 2.2E–16), and magenta (*R* = 0.58, *p* = 2.2E–16) modules of RAA, suggesting that the modules were strongly associated with AF.

### Differential Expression Profiles and Enrichment Pathways of LAA and RAA

**FIGURE 5:**
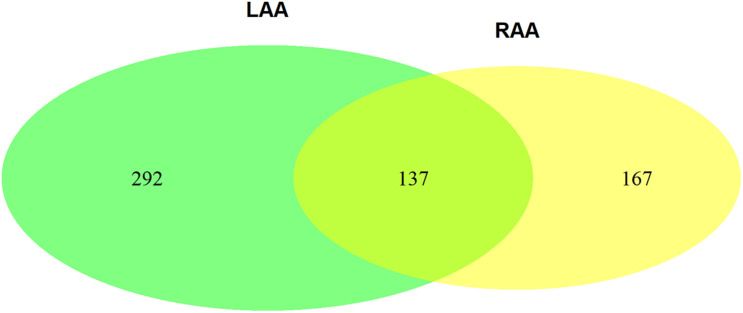
The Venn plot constructed by two AF-associated modules in LAA and three AF-associated modules in RAA (GS > 2). GS, gene significance; LAA, left atrial appendage; RAA, right atrial appendage.

**FIGURE 6:**
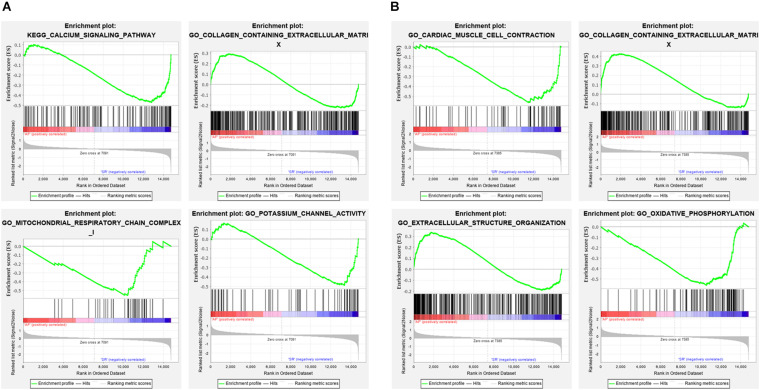
The enrichment pathways from GSEA. **(A)** The enrichment pathways from GSEA in LAA. **(B)** The enrichment pathways from GSEA in RAA. LAA, left atrial appendage; RAA, right atrial appendage.

The Venn plot constructed by five modules associated with AF showed 429 and 304 genes with high propensity to aggregate, respectively, in LAA and RAA (Figure 5). However, only 137 genes were overlapped between LAA and RAA, suggesting the difference of expression profiles of left and right hearts for AF. In addition, in LAA, GSEA showed that the transcriptome data were enriched in the pathways such as calcium signaling pathway (*p* = 0, FDR = 0.017), collagen containing extracellular matrix (*p* = 0, FDR = 0.2), mitochondrial respiratory chain complex (*p* = 0.002, FDR = 0.01), and potassium channel activity (*p* = 0, FDR = 0.02) (Figure 6A). In RAA, data demonstrated that the pathways such as cardiac muscle cell contraction (*p* = 0, FDR = 0.002), collagen containing extracellular matrix (*p* = 0, FDR = 0.0005), extracellular structure organization (*p* = 0, FDR = 0.17), and oxidative phosphorylation (*p* = 0, FDR = 0) were enriched (Figure 6B). These results suggest that the pathogenesis of AF is related to the factors such as the increase of atrial fibrosis, the decrease of channel protein function, and mitochondrial dysfunction. All GSEA-related data are included in Supplementary File 2.

### Determination of Newly AF-Associated Biomarkers

**FIGURE 7:**
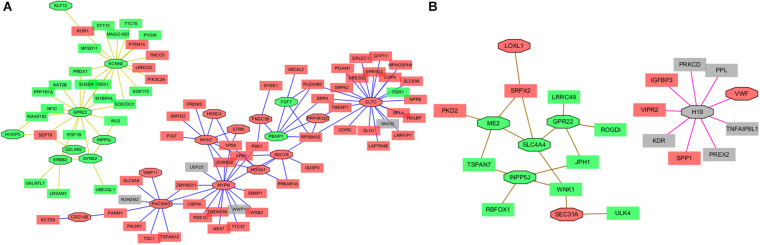
The Network view from Cytoscape. **(A)** The Network view from Cytoscape in LAA. **(B)** Network view from Cytoscape in RAA. The module edge > 0.15. Green color represents downregulated genes with GS > 2, red color represents upregulated genes with GS > 2, and gray color represents the genes with GS < 2. Octagon nodes represent core genes. GS, gene significance; LAA, left atrial appendage; RAA, right atrial appendage.

**FIGURE 8:**
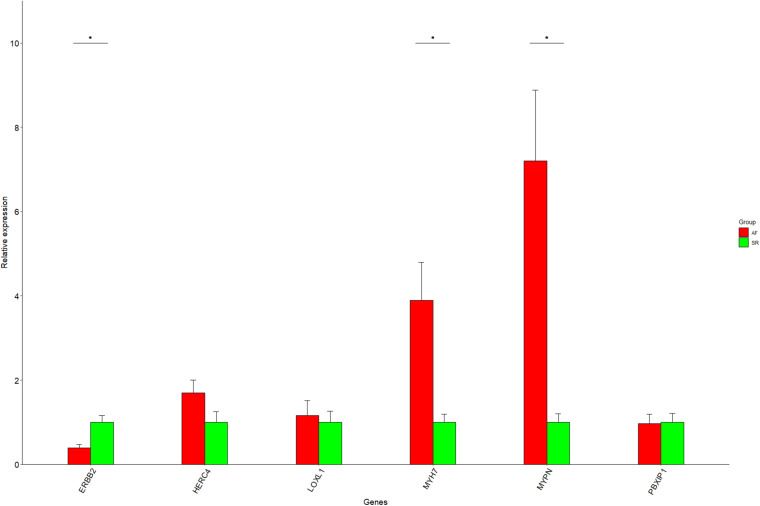
The RT-qPCR results for six genes. Data are shown as mean ± SE, and each sample is in triplicate. AF, atrial fibrillation; SR, sinus rhythm. *P < 0.05.

One of the advantages of Cytoscape is the ability to intuitively and visually demonstrate connections between genes with association evidence by TOM. In LAA, the edge file “Blueyellow.edges” (Supplementary File 3) reflecting the weight relationships between the blue plus yellow module genes was obtained by the “exportNetworkToCytoscape” function in WGNNA package and was as an input file of Cytoscape. Setting the weight cutoff value of the edge to 0.15, we observed 130 genes crosslinked with each other in the network, and 22 core genes (the octagon nodes) are presented in Figure 7A. By the GWAS test set, the AF-associated gene *MYH7* (adj *p*_dis_ = 7.6E–6, adj *p*_test_ = 0.014, GS = 3.95) confirmed in previous studies was identified, while four novel biomarkers associated with AF were determined, including *ERBB2* (adj *p*_dis_ = 5.1E–7, adj *p*_test_ = 0.013, GS = 3.34), *HERC4* (adj *p*_dis_ = 6E–6, adj *p*_test_ = 0.0042, GS = 3.0), *MYPN* (adj *p*_dis_ = 0.0004, adj *p*_test_ = 0.02, GS = 2.07), and *PBXIP1* (adj *p*_dis_ = 0.00036, adj *p*_test_ = 0.0002, GS = 3.3). Following the same steps mentioned previously (the edge file “Yellowbrowmagenta.edges” seen in Supplementary File 4), we just obtained eight core genes and eventually get one AF-associated gene *LOXL1* (adj *p*_dis_ = 0.047, adj *p*_test_ = 0.07, GS = 3.0) in RAA (Figure 7B). Taken together, these results could indicate that the triggering and maintenance of AF is incline to the left atrium. Subsequently, the six AF-associated loci were verified from the LAA of 30 clinical patients by RT-qPCR, and the data showed that the expression of *ERBB2*, *MYH7*, and *MYPN* in AF group had significant change compared with that in SR group, while that of *HERC4*, *LOXL1*, and *PBXIP1* had no change (Figure 8).

## Discussion

**FIGURE 9:**
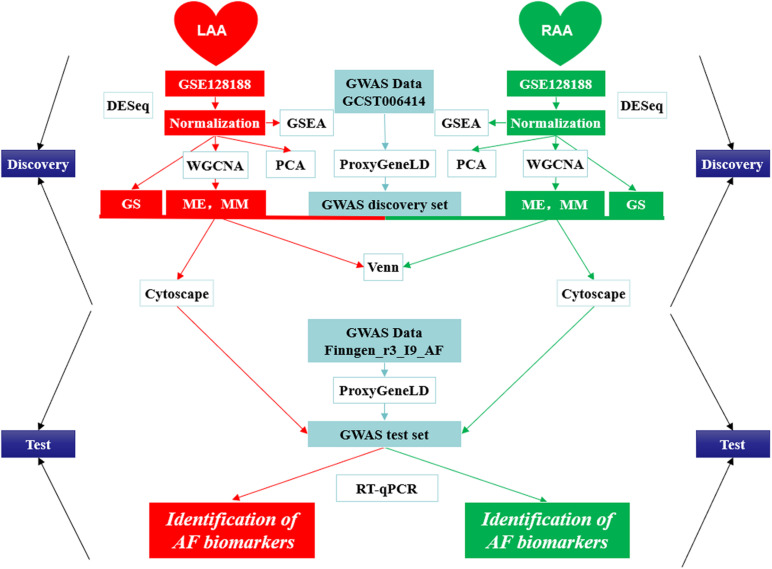
The design scheme of this study. GS, gene significance; LAA, left atrial appendage; ME, module eigengene; MM, module membership; PCA, principal component analysis; RAA, right atrial appendage.

In the present study, we first combined GWAS with gene expression profile data from the LAA and RAA of patients to dig newly AF-associated biomarkers by ProxyGeneLD and WGCNA. In LAA and RAA, five AF-associated modules were identified and were verified by the relationships between MM and GS. In the GWAS discovery set, 30 core genes which exhibited GWAS nominal evidence for AF associations (adj *p*_dis_ < 0.05) were visually shown in the network constructed by Cytoscape, and six AF-associated genes were determined (LAA: 5 and RAA: 1) by the GWAS test set. Using RT-qPCR, two novel genes associated with AF *ERBB2* and *MYPN* are eventually identified. The scheme of this study is shown in Figure 9 and other WGCNA-related data in this study are illustrated in Supplementary File 5.

In recent years, some biomarkers associated with AF have been successfully identified by GWAS. However, the genetics of AF risk is still not fully elucidated, particularly the subtle-effect genes associated with AF (*p* > 5E–08). Although network-based analyses such as GO and KEGG can be applied to identify subtle-effect genes, they do not consider the mutual interconnection between genes (Ritchie, 2009; Torkamani and Schork, 2009). Farber (2013) combined GWAS with WGCNA to explore network modules and new biomarkers relative to osteoporosis, which inspired the implementation of the analysis for AF, with some modification. In this study, we applied the combination of GWAS with expression profile data on AF and obtained useful network modules and core genes by taking into account unsupervised clustering, flexible soft thresholds, the connections between genes, and the reduced dimensionality of thousands of expressed genes. The combination of ProxyGeneLD and WGCNA may provide a new perspective for revealing more hidden biomarkers associated with AF.

Using WGCNA, our results repeated most of the differentially expressed genes in the original literature (Thomas et al., 2019) (the upregulated/downregulated in LAA: *KCNJ2* and *PRKAR1A*/*FGFR2* and *PTPRU*, the upregulated/downregulated in RAA: *COL12A1* and *RPL3L*/*CADM2* and *COG5*, and the simultaneously expressed in LAA and RAA: the *GPR22*, *PPP1R1A*, and *RGS6*). Similarly, the results of pathway analysis were also reproduced in the original literature, such as cation channel and plasma membrane in LAA, and collagen-, extracellular matrix-, clathrin-, and Golgi-related pathways in RAA. The aforementioned data suggest the reliability and repeatability of WGCNA and GSEA. Besides, our results showed that multiple pathways related to AF were not present in the original text, including calcium signaling pathway, collagen-containing extracellular matrix, mitochondrial respiratory chain complex, and potassium channel activity in LAA, most of which are complicated in either atrial structural or electrical remodeling (Jalife and Kaur, 2015), suggesting that GSEA taking into account gene expression values could have an advantage over classic enrichment pathway software such as GO or KEGG.

Based on the study protocol published by Farber (2013), with small modifications, we mined potential AF-associated genes using GWAS and transcriptome expression profile data on left–right atrial appendages, and the six confirmed and newly AF-associated biomarkers were identified and validated. Previous studies have reported that in atrium the upregulated *MYH7*, a ventricular-myosin heavy chain isoform, are associated with AF, which might improve economy of contraction by the increase in metabolic demand of AF (Barth et al., 2005). In our study, the significant expression elevation was observed for this gene, not only in our data-mining process using bioinformatics but also in our experimental verification using RT-qPCR. Although the adj *p*_test_ value of *LOXL1*, as the only candidate marker of RAA associated with AF in this study, is greater than 0.05, it plays a key role in extracellular matrix homeostasis and remodeling, family members of which participate in heart failure, myocardial infarction, cardiac hypertrophy, dilated cardiomyopathy, and AF (Philp et al., 2018; Rodriguez and Martinez-Gonzalez, 2019). It was not observed that the expression of this gene changed in human LAA by RT-qPCR, similar to our data-mining result. However, whether the gene is differentially expressed in human RAA still needs to be further verified by subsequent experiments.

In this study, the four AF markers, *ERBB2*, *HERC4*, *MYPN*, and *PBXIP1*, have not been reported in previous studies. *ERBB2*, associated with heart failure, can improve mammalian heart regeneration, and promote cardiomyocyte dedifferentiation and proliferation (D’Uva et al., 2015). Belmonte et al. (2015) reported that its overexpression can upregulate antioxidant enzymes, reduce basal levels of reactive oxygen species, and thus protect the myocardium. Our data mining and RT-qPCR results showed that the expression level of *ERBB2* is downregulated, indicating that the gene may increase reactive oxygen species, decrease antioxidant enzymes, and diminish the ability of cardiomyocytes to regenerate, and thus that it could be one of AF remodeling substrates. *MYPN* as a messenger gene links structural and gene regulatory molecules to the nucleus in cardiomyocyte by translocation from the I-bands or Z-disk. It is clear that numerous mutation loci in the gene are associated with hypertrophic, dilated, and restrictive cardiomyopathy (Chen et al., 2017). One study demonstrated that *MYPN* knockout mice exhibited a 48% reduction in myofiber cross-sectional area and significantly increased fiber number, compared with wild-type controls (Filomena et al., 2020). Our results showed that this gene is upregulated, suggesting that it may induce abnormal atrial myocardial fibrosis and therefore be associated with the occurrence of AF. Furthermore, our data from RT-qPCR showed that *HERC4* as a tumor suppressor (Xu et al., 2019) and *PBXIP1* (Arrington et al., 2012) contributing to tumor cell growth and migration are not related to AF, and further studies are needed.

Because there is only a small amount of genome-wide expression profile data available for AF in public databases, especially containing both LAA and RAA of individuals, the statistical efficacy may be limited in this study. However, studies using WGCNA to analyze small sample data are comparatively abundant, and convincing results have been obtained from previous studies (Gargalovic et al., 2006; Gong et al., 2007; Farber, 2013), and our data-mining results were confirmed by RT-qPCR. In addition, although no stratification of clinic types of AF was conducted in our study, we expected that this problem be solved using a larger sample in the future. Furthermore, the lack of RAA samples limits our verification of AF markers in RAA, which will be improved in subsequent studies. However, as a powerful bioinformatic method, coexpressed nodes of WGCNA may have interdependent mechanistic relationships that are not yet appreciated, which may lead to the co-identification of genes in associated studies (Liu et al., 2015a). Of note, the present study did not identify all the genes that were known in the previous GWAS, as it was only a supplementary work for exploring biomarkers related to AF.

## Conclusion

In summary, we revealed effectively two newly AF-associated genes *ERBB2* and *MYPN* by integrating GWAS with expression profile data using theories and methods of systems biology, and based our hypothesis on the missing heritability generated by GWAS data. These findings highlight the value of the network approach in the acquisition of newly AF-associated genes and provide insights into the pathological mechanisms of AF.

## Data Availability Statement

The datasets presented in this study can be found in online repositories. The names of the repository/repositories and accession number(s) can be found in the article/Supplementary Material.

## Ethics Statement

The studies involving human participants were reviewed and approved by The IRB of Zhengzhou No. 7 People’s Hospital. The patients/participants provided their written informed consent to participate in this study.

## Author Contributions

XM and YY: study conception and design, analysis of data, and drafting of manuscript. JZ: interpretation of data. YN, KW, and CF: acquisition of data, sample collection and testing, and critical revision. All authors read and approved the final manuscript.

## Conflict of Interest

The authors declare that the research was conducted in the absence of any commercial or financial relationships that could be construed as a potential conflict of interest.

## Footnotes

1 https://console.cloud.google.com/storage/browser/finngen-public-data-r3/summary_stats/
